# The Simplified Medication Adherence Questionnaire: validation of a Brazilian-Portuguese version in hypertensive adults

**DOI:** 10.3389/fphar.2024.1348917

**Published:** 2024-04-11

**Authors:** Simony M. Soares, Mirela Q. de Almeida Diniz, Dilcy Morgana B. M. C. Davino, Fernanda B. Albieri, Adriano S. Santos, Elisdete M. S. Jesus, Divaldo P. Lyra-Junior, Sabrina J. Neves, Alfredo D. Oliveira-Filho

**Affiliations:** ^1^ Department of Pharmacy, Laboratory of Teaching and Research in Social Pharmacy (LEPFS), Federal University of Sergipe, São Cristóvão, Brazil; ^2^ Pharmacotherapy Research Group, Institute of Pharmaceutical Sciences, Federal University of Alagos, Maceió, Brazil; ^3^ Faculty of Pharmaceutical Sciences, State University of Campinas, Campinas, São Paulo, Brazil

**Keywords:** hypertension, Simplified Medication Adherence Questionnaire, patient-reported outcome measures (MeSH), validation studies (MeSH), psychometrics (MeSH), medication adherence (MeSH), treatment adherence and compliance

## Abstract

**Background:**

Self-reported adherence scales are widely used in research and practice because they are low in cost and easy to apply. A free version in Brazilian-Portuguese of the Simplified Medication Adherence Questionnaire (SMAQ) can be a useful alternative for determining the adherent behavior of hypertensive patients.

**Purpose:**

To translate and evaluate the psychometric properties of the Brazilian-Portuguese version of the SMAQ therapeutic adherence scale for patients with arterial hypertension.

**Patients and methods:**

A multicenter, cross-sectional study was conducted in five outpatient units in Maceió-AL and Aracaju-SE between January and July 2019. A total of 117 patients aged over 18 years using antihypertensive drugs were recruited. The cross-cultural adaptation followed international methodological recommendations. Internal consistency (Cronbach’s alpha) was tested as a reliability parameter. Criterion and construct validity were verified by concurrent validation, exploratory factor analysis (EFA), and validation by known groups.

**Results:**

The participants had a mean age of 56.6 years (SD = 10.7 years); most were female (72.6%). The mean number of antihypertensives prescribed per patient was 1.87 (SD = 0.87). There were 79.5% (*n* = 86) of patients considered non-adherent. Internal consistency was satisfactory (Cronbach’s alpha = 0.63). A satisfactory correlation coefficient was verified with the Morisky–Green–Levine test as an external criterion (*r* = 0.56, *p* < 0.001). The scale’s sensitivity measured through known group validity was 75.3%, specificity 29.5%, positive predictive value 63.9%, and negative predictive value 41.9%. We identified two factors of the instrument’s construct from EFA: specific medication-taking behaviors and barriers to adherence. The initial KMO measure of sampling adequacy was 0.691, and Bartlett’s test of sphericity was significant (χ^2^ = 118.342, *p* < 0.001).

**Conclusion:**

The Brazilian-Portuguese version of the SMAQ scale proved valid and reliable for determining adherence to the pharmacotherapy in hypertensive patients. It showed more ability to detect non-adherent patients but with low specificity, possibly influenced by high social desirability.

## Background

Arterial hypertension is the main preventable cause of cardiovascular disease (CVD) and is the most important risk factor for death and inability globally. It affects more than 1.2 billion people aged between 30 and 79 years, of whom approximately 82% live in low- and middle-income countries (NCD Risk Factor Collaboration NCD-RisC, 2021). In Brazil, more than 300 thousand deaths were related to cardiovascular complications in 2019 (PAHO, 2021). Although much evidence shows that decreased blood pressure decreases early morbimortality, blood pressure control rates are low worldwide (Williams et al., 2018; Brouwers et al., 2021; NCD Risk Factor Collaboration NCD-RisC, 2021; Kario et al., 2022).

Pharmacotherapy adherence is vital to achieving therapeutical goals and disease control (Zayed et al., 2019). Recently, however, an increasing number of patients seem to resist to antihypertensive treatment. It is estimated that only 23% of women and 18% of men with hypertension present adequate blood pressure control. Part of this result is related to low adherence to prescribed pharmacotherapy (Judd and Calhoun, 2014; Ewen et al., 2015; Durand et al., 2018; NCD Risk Factor Collaboration NCD-RisC, 2021). This has been a challenge to health professionals and is an important causal factor of arterial hypertension treatment failure.

Non-adherent behavior of patients can be influenced by a series of predictive factors, intentional or not, such as beliefs, disease status, forgetfulness, limited health literacy, and socio-economic factors (Nguyen et al., 2014; Zayed et al., 2019). Different direct and indirect methods of therapeutic adherence evaluation could help identify adherent behaviors and more effective treatment management, thus reducing cardiovascular adverse event risks. Each method has advantages and disadvantages, and there is thus no agreement on a single gold standard approach. While direct methods are more accurate, they are difficult to apply in real life and cost more (Beyhaghi et al., 2016).

The most used indirect methods comprise patient self-reporting, pill counts, and pharmacy refills. Although these methods could overestimate adherence and have low accuracy and sensitivity, self-reporting is the most used method as it shows good cost/efficiency and cost/time relationships, and it is easy to apply in practice and research with large populations. Furthermore, self-reporting is the only method capable of determine the reasons that lead patients to certain behaviors (Ben et al., 2012; Unni and Farris, 2015; Gellad et al., 2017).

Ideally, pharmacotherapy adherence scales should be easy to apply and should correctly identify not only medicine intake behavior but also the main barriers to adherence. In addition, it is also preferable that these scales are free, summary, accessible to clinical practice, and show good psychometric properties. One of the most used self-reporting instruments is the Morisky Medication Adherence Scale (MMAS-8), which was validated in Brazilian-Portuguese in 2013 for application to hypertensive patients (Morisky et al., 2008; Oliveira-Filho et al., 2014). However, in recent years, this scale has demanded acquisition of a license, restricting its usefulness—especially in the public health system’s clinical practice.

An alternative, the Simplified Medication Adherence Questionnaire (SMAQ) scale, originally developed to evaluate the adherent behavior of people living with HIV (Knobel et al., 2002), comprises aspects of medicine intake behavior and adherence barriers and thus could easily be used for distinct chronic diseases, such as hypertension. Beyond short-term application, the scale showed good validation levels, with favorable sensitivity and specificity results for the studied conditions (Nguyen et al., 2014).

In this context, the present study aimed to cross-culturally adapt to the Brazilian-Portuguese language, analyze the psychometric properties, and identify whether the SMAQ is suitable for evaluating pharmacotherapy adherence in hypertensive patients.

## Methods

A translation and cross-cultural adaptation study was conducted from April to November 2018 to obtain a Brazilian-Portuguese version of the SMAQ, followed by a psychometric property evaluation study of instruments conducted from January to August 2019.

**FIGURE 1:**
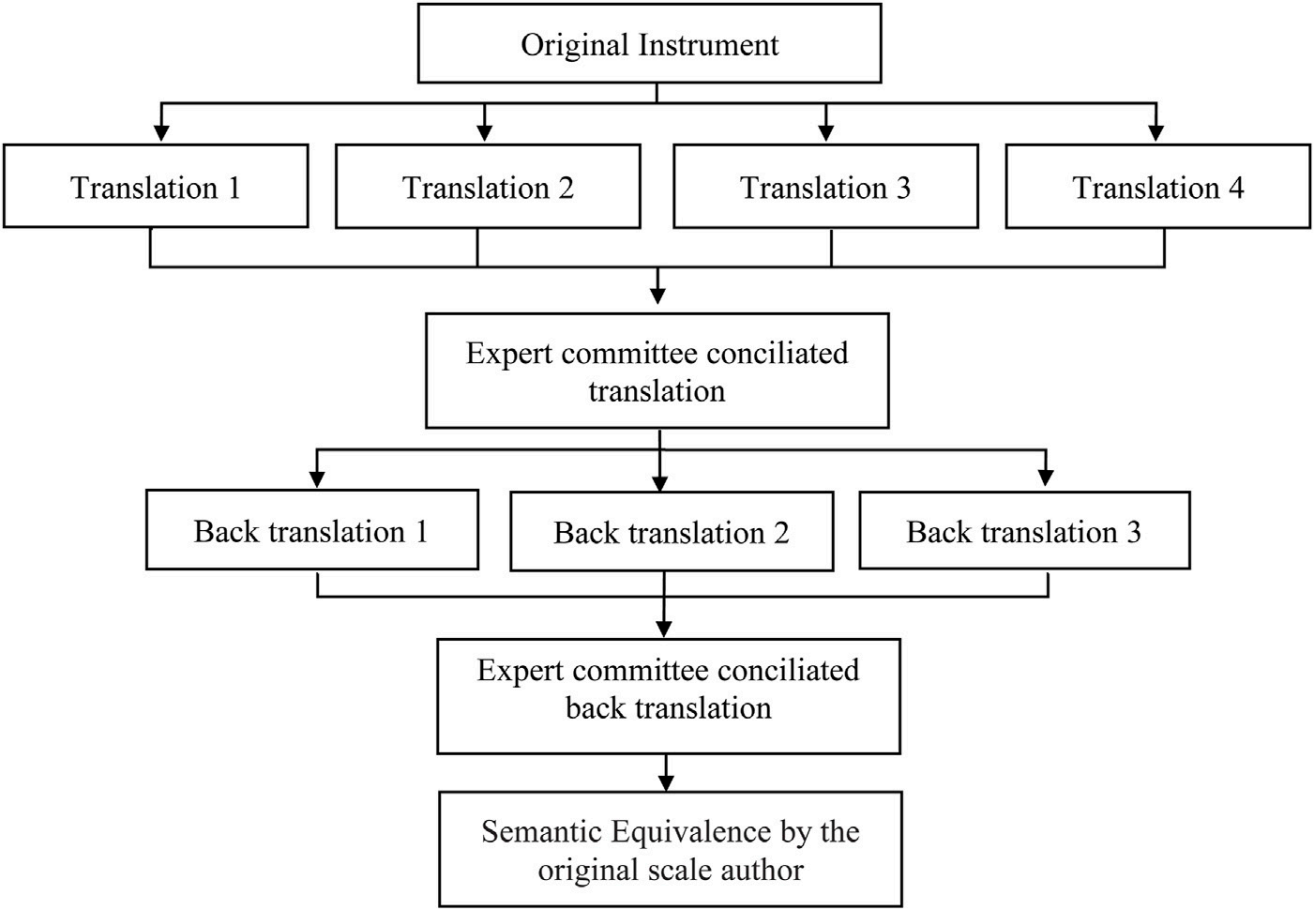
Proceedings of cross-cultural adaptation of instruments.

For the translation and cross-cultural adaptation phase, this study followed the international methodological recommendations for the cross-cultural adaptation of self-reporting parameters, which advocate the following combined steps: translation, back-translation to original language, translation summary, semantic equivalence of translations, pretest, and psychometric property evaluation (Figure 1) (Guillemin et al., 1993; Beaton et al., 2000; Wild et al., 2005; Ferreira et al., 2014).

Permission to translate and cross-culturally adapt the SMAQ instrument was obtained by contacting the original version’s author, Dr. Hernando Knobel of the Internal Medicine Department, del Mar Hospital, Barcelona, Spain. This study was approved by the Federal University of Sergipe Research Ethics Committee under CAAE 92716418.1.0000.5546.

### Cross-cultural adaptation steps

#### Instrument translation

First, four bilingual translators who were Brazilian-Portuguese speakers and English-proficient independently translated the original version of SMAQ into Brazilian-Portuguese. Two of them did not know about the study theme, and the other two were informed about the objectives and content of the study. The translators were selected by convenience and invited by email. A 30-day deadline was agreed for returning the translations. To synthesize the results, an expert committee formed by three research pharmacists who had good Portuguese knowledge and English proficiency compared the four scale translations (SMAQ versions A1, A2, A3, and A4).

Ambiguities or discrepancies in translated words were resolved by consensus, leading to a single translated version of the instrument (version A0).

#### Back-translation

In this step, the Brazilian-Portuguese version A0 was back-translated into the original English by three other bilingual translators whose native language is English and who operated in a Brazilian-Portuguese domain. The back translators did not receive information about the study objectives or concepts. Therefore, we hired English-native individuals, two of whom were resident in Brazil, who work in English–Portuguese and Portuguese–English translation to do the back-translations. All three back-translated SMAQ versions (versions RA1, RA2, and RA3) were again compared by an expert committee formed by the previous three researchers and another research pharmacist with a master’s degree in pharmaceutical science and who was bilingual (Brazilian-Portuguese and English fluent). Ambiguities or discrepancies were resolved by consensus, leading to the instrument’s back-translation (version RA0). These back-translation versions were submitted to the instrument’s original version developers to obtain semantic equivalence to the translated versions. The expert committee confirmed idiomatic, experiential, and conceptual equivalence (prefinal versions).

We conducted two cycles of pretesting. In the first, 15 individuals were recruited and answered the prefinal SMAQ version that the expert committee previously defined. In the second cycle, we applied the scale once more with five participants.

### Psychometric property evaluation

#### Study design

The pretest and psychometric property evaluation steps were conducted following the descriptive cross-sectional cohort study model.

#### Study site

This was a multicentric study. Data collection of the pretest phase was conducted in the cardiology clinic of a teaching hospital in Aracaju, Sergipe. For the validation phase, as well as the already mentioned site, the study was simultaneously conducted in two units of family health and two public state institutions that provide pharmaceutical care in Maceió, Alagoas.

#### Study population

The study population included patients with confirmed systemic arterial hypertension aged over 18 years, who were using antihypertensive drugs for at least 30 days. We adopted, as exclusion criteria, the use of antihypertensive drugs to treat other health conditions.

#### Data collection

The data were collected by researchers, pharmacy graduate students, and properly trained pharmacists through patient interviews and blood pressure measures. Pretest data were collected between September and November 2018. Data for the validation phase were collected between January and July 2019. The interviews were based on four questionnaires: the first instrument collected sociodemographic data (name, register number, age, sex, and schooling) and history of antihypertensive drug use; the instruments following were the SMAQ pharmacotherapy adherence scale in the final translated versions; a Brazilian-Portuguese version of the Morisky–Green–Levine (MGL) scale to concurrent validation (Morisky et al., 1986; BEN, 2011); a Brazilian version of the Marlowe–Crowne social desirability scale (MC-SDS-BR) (Crowne and Marlowe, 1960; Ribas et al., 2004). Systolic (SBP) and diastolic (DBP) blood pressure values were obtained using calibrated manual sphygmomanometers, taking the average of two measures with at least a 1-min interval between measures, as advocated by the seventh Brazilian Guideline for Arterial Hypertension (Malachias, 2016). In the present study, a complete case (CC) analysis was performed via casewise deletion.

#### Medication adherence

Responses to the SMAQ items were coded analogous to the original version (Knobel et al., 2002). Patients were considered non-adherent if they gave positive responses to any of the qualitative questions (1, 2, 3, and 5), had missed more than two doses over the past week, or had missed over 2 days of total non-medication during the past 3 months.

The SMAQ contains items that elicit information on i) specific medication-taking behaviors (e.g., dose taken and dose frequency) and ii) barriers to adherence (e.g., forgetfulness and side effects) (Nguyen et al., 2014). According to Krousel-Woord et al (2021), these explicit attitudes influence health behavior that is consciously chosen or premeditated, and they are usually assessed through self-report surveys that focus on patients’ conscious attitudes toward disease and its treatment.

#### Pretest

In this step, the researcher and two previously trained research pharmacist collaborators applied the prefinal versions of the translated instrument to a 20-individual group from the target population using the methodological adjustment proposed by Ferreira et al. (2014). We thus conducted two cycles of pretesting. In the first, individuals were recruited and answered the prefinal SMAQ version that the expert committee had previously defined. In the second cycle, we applied the scale again.

All individuals who consented to participate in the study had been informed about the objectives and nature of the research and were asked to sign the Consent Informed Test, in accordance with CNS resolution nº 466/2012 (BRASIL, 2012).

The pretest was conducted as a pilot study, according to the item (data collection) and the guide to apply the SMAQ adherence scale. If any individual in this phase had a question about or difficulty understanding the instrument items, the question would be registered in the corresponding scale formulary to be discussed again by the expert committee and possibly reformulated.

### Psychometric property evaluation

#### Internal consistence

To verify reliability by internal consistency, we used Cronbach’s alpha, which indicates if each item of a scale is appropriated for evaluating the nominated concept (Cronbach, 1951). On the other hand, α is a function of the interrelation of items with the number of items, so the number of items is a factor which affects the α coefficient. In simple terms, it is a reliability statistical parameter from 0 to 1 where values above 0.6 may be considered satisfactory and those exceeding 0.8 may indicate high internal consistency (Taherdoost et al., 2014; Ventura-León and Peña-Calero, 2021; Zakariya, 2022). In this study, Cronbach’s alpha values under 0.5 were considered unacceptable. We also evaluated the corrected item–total correlation and Cronbach’s alpha if the item was excluded to verify the contribution of each item to the general reliability coefficient of each scale.

#### Criterion validation

The criterion validation (concurrent validation) of the SMAQ scale was evaluated through the Morisky–Green–Levine (Morisky et al., 1986; BEN, 2011) test association to determine the scales’ correlation coefficient. R-values above 0.5 were considered satisfactory and above 0.7 a strong correlation (Drost, 2011; SCHMIDT and DANTAS, 2011; Souza et al., 2017). The considered significance level was *p* < 0.05. The instrument has four questions structured as dichotomous “yes/no” answers. The worst adherence is considered to be many “yes” answers; only all negative answers classify the patient as an “adherer.”

In the analysis of per-item correlation, the association between each individual item of the SMAQ scale and the total score of the MGL criterion was evaluated. This involved calculating Pearson’s correlation coefficient for each item of the SMAQ scale in relation to the total MGL score.

#### Factor analyses

Exploratory factor analysis (EFA) was conducted on the SMAQ tool. The Kaiser–Meyer–Olkin (KMO) measure of sampling adequacy and Bartlett’s test of sphericity were used to determine database eligibility. Visual inspection for an inflection point in scree plots was utilized to identify initial factors. EFA was conducted using principle components analysis with direct Oblimin rotation; factor loadings >0.30 were considered valid (Taherdoost et al., 2014).

#### Validation by known groups

In this evaluation, we tested the capability of a measure to make distinctions among groups of individuals that differ according to some known factor. The construct validation by known groups was conducted by associating the evaluated scales with blood pressure control (SBP <140 mmHg and DBP <90 mmHg) using the chi-squared and Student’s t-tests and considering that patients with low adherence scores also present low BP control. The significance level was considered to be *p* < 0.05.

The sensitivity, specificity, and positive and negative predictive value properties were determined to verify whether the Brazilian-Portuguese version of the SMAQ scale would serve as a screening tool to identify patients with low BP control.

In addition in this step, social desirability was compared among adherent and non-adherent patients using the Marlowe–Crowne Social Desirability Scale (Crowne and Marlowe, 1960; Ribas et al., 2004).

### Sample size

A total of 117 patients with arterial hypertension, predominantly attending outpatient units of the Unified Health System, were selected using non-probabilistic convenience sampling. The minimum sample sizes required to achieve a “very good” quality rating for various measurement properties, as outlined in the COSMIN Study Design Checklist for Patient-Reported Outcome Measurement Instruments, were also considered. Specifically, ≥50 patients were required for criterion validity, while ≥100 patients were necessary for internal consistency, reliability, and construct validity (Mokkink et al., 2019).

### Data statistical analysis

Data analysis was conducted using the SPSS statistical analysis program version 25.0. The statistical analyses were Cronbach’s alpha to verify internal consistence, descriptive analysis, Student’s t-test, chi-squared, and the Spearman coefficient correlation to test the hypothesis and relationships among pharmacotherapy adherence and other independently variables. Significance was considered when *p* < 0.05.

### Ethical aspects

All individuals directly involved who consented to participate in this study were previously informed about the objectives and nature of this research and signed the Consent Informed Term in accordance with CNS resolution nº 466/2012 (BRASIL, 2012).

## Results

### Cross-cultural adaptation of the pharmacotherapy adherence scale

#### SMAQ scale translation

Table 1 compares the translated versions and the synthesis of the SMAQ instrument translations. In the first two versions (A1 and A2), translators who did not know about the study theme translated the scale using less formal English than versions A3 and A4, which were made by translators who received guidance about the study objectives. However, in version A1, it is possible to observe more frequently the use of technical terms, common to the health field, such as “*medicamento”* (medicine) instead of *“remédio”* (pills) when the translator was a health professional (nutritionist).

**TABLE 1:** Original version items, translations, and the translation synthesis version of the SMAQ instrument.

SMAQ original version	Translation				Translation synthesis version A0
	Version A1	Version A2	Version A3	Version A4	
1- Do you ever forget to take your medicine?	Você esquece de tomar seus remédios?	Você alguma vez esquece de tomar oremédio?	O(A) senhor(a) frequentemente esquece de tomar seu(s) medicamento(s)?	Você esquece de utilizar seu(s) medicamento(s)?	Você esquece de tomar o(s) seu(s) medicamento(s)?
2- Are you careless at times about taking your medicine?	Você, às vezes, é descuidado para tomar seus medicamentos?	Você as vezes descuida de tomar o remédio?	O(A) senhor(a), às vezes, é descuidado quanto à tomada de seu(s) medicamento(s)?	Você se distrai quando utiliza seu(s) medicamento(s)?	Você, às vezes, é desatento para tomar o(s) seu(s) medicamento(s)?
3- Sometimes if you feel worse, do you stop taking your medicines?	Se você sente piora, você para de tomar os medicamentos?	Às vezes, se você não se sentir bem, para de tomar o remédio?	O(A) senhor(a), às vezes, para de tomar seu(s) medicamento(s) ao sentir-se pior?	Quando se sente pior, você para de utilizar seu(s) medicamento(s)?	Às vezes, quando se sente pior, você para de tomar seu(s) medicamento(s)?
4- Thinking about the last week. How often have you not taken your medicine?	Pensando na última semana. Quantas vezes você não tomou seus medicamentos?	Considerando a semana passada, quantas vezes você não tomou o remédio?	Na última semana. Com que frequência o(a) senhor(a) deixou de tomar seu(s) medicamento(s)?	Pensando na semana passada, quantas vezes você deixou de utilizar seu(s) medicamento(s)?	Pensando na última semana. Quantas vezes você deixou de tomar seu(s) medicamento(s)?
5- Did you not take any of your medicine over the past weekend?	Você deixou de tomar algum de seus medicamentos durante a última semana?	Você deixou de tomar algum de seus remédios no fim de semana passado?	O(A) senhor(a) deixou de tomar todos os seu(s) medicamento(s) no último fim de semana?	Você deixou de utilizar algum medicamento final de semana passado?	Você deixou de tomar algum de seus remédios no fim de semana passado?
6- Over the past 3 months, how many days have you not taken any medicine at all?	Nos últimos três meses, quantos dias você não tomou nenhum dos seus medicamentos?	Nos últimos três meses, quantos dias você deixou de tomar os remédios?	Nos últimos três meses, quantos dias o(a) senhor(a) deixou de tomar todos o(s) medicamento(s)?	Nos últimos três meses, quantos dias você ficou sem utilizar algum medicamento?	Nos últimos três meses, quantos dias você não tomou nenhum dos seus medicamentos?

In the translation conciliation process, the expert committee decided to mostly use the term *“medicamento”* (medicine), judging it to be technically more appropriate in a global context and preserving the original meaning. Nevertheless, they decided to use the term *“remédio”* (pills) in item 5 to approximate it to patients’ informal language, considering that one of the aims of the study is to make the scale accessible to users of Brazil’s public health system, who usually have low health literacy.

#### Instrument back-translation

In the next step of the SMAQ instrument adaptation—the back-translation—the RA0 version was translated back into English by another three translators, independent of each other, to verify whether the Brazilian versions had any important mistakes or translation inconsistencies that could make the actual content different from the original. As foreseen in the methods, the back-translators were not previously informed about the study objectives.

After this process, a new version conciliation step was conducted by an expert committee which included a fourth expert who was English-proficient to increase the synthesis reliability.

#### The SMAQ scale back-translation

Table 2 shows the original SMAQ scale followed by the back-translations and the version synthesis. The original scale author, Dr. Hernando Knobel, confirmed the semantic equivalence without needs of adjustment.

**TABLE 2:** Original version items, back-translations, and back-translation synthesis version of the *SMAQ* instrument.

SMAQ	Back-translation			Back-translation synthesis version RA0
Original version	Version RA 1	Version RA 2	Version RA 3	
1- Do you ever forget to take your medicine?	Do you ever forget to take your medication(s)?	Do you forget to take your medication(s)	Have you ever forgotten to take your medication(s)?	Do you forget to take your medication(s)?
2- Are you careless at times about taking your medicine?	Do you sometimes skip doses of your medication(s)?	Are you sometimes careless about taking your medication(s)	Are you always aware of your medication(s) needs when taking your medication?	Are you sometimes careless about taking your medication(s)?
3- Sometimes if you feel worse, do you stop taking your medicines?	If you feel worse, do you sometimes stop taking your medications(s)?	Sometimes, when you feel worse, do you stop taking your medication(s)?	Have you stopped taking your medication when you start feeling worse?	If you feel worse, do you sometimes stop taking your medications(s)?
4- Thinking about the last week. How often have you not taken your medicine?	Considering last week, how many times did you not take your medication(s)?	Thinking about the last week, how many times have you not taken your medication(s)	How many times did not you take your medication during the last week?	Thinking about the last week, how many times have you not taken your medication(s)?
5- Did you not take any of your medicine over the past weekend?	Did you stop taking your medication(s) last weekend?	Did you not take any of your medicines last weekend?	Did you not take any of your medication during the last weekend?	Did you not take any of your medication(s) last weekend?
6- Over the past 3 months, how many days have you not taken any medicine at all?	During the last 3 months, how many days have you not taken any of your medication(s)?	In the last 3 months, on how many days did you not take any of your medication(s)?	In the last 3 months, how many days have you not taken your medication?	During the last 3 months, how many days have you not taken any of your medication(s)?

#### Pretest

The pretest phase was the last step of the semantic equivalence process and was the pilot model of the validation process. The 20 participants attended individually. Their mean age was 57 years (22–79 years, standard deviation = 14.6); 65% were female, half did not complete middle school, 85% affirmed having some comorbidity, and 65% had had an arterial hypertension diagnosis for more than 5 years. As for the number of antihypertensive drugs in use, half of participants affirmed using three or more drugs, which should be an alert for possible resistant hypertension.

Only two participants agreed to self-apply the scales, which may be associated with the fact that 50% of participants had low health literacy, which led the researchers to apply the scales by interviewing participants as follows:

In the first of two cycles of pretesting (with 15 and 5 participants, respectively), approximately 30% of the initial sample had some difficulty understanding the term “*desatento*” (“careless”) in the SMAQ scale item 2. This issue was brought to the attention of the expert committee, who decided to replace the term with “*descuidado*” (“inattentive”). In the second cycle, after adjustments, the lack of clarity with the new term still remained. Therefore, the expert committee reverted to the original term (“careless”) and approved the final version for further reliability and validation tests to determine whether additional adjustments were necessary.

### Psychometric properties evaluation

#### Sociodemographic data and pharmacotherapy adherence

The Brazilian-Portuguese final version of the SMAQ scale was applied to 117 outpatients with arterial hypertension diagnosis. The mean age was 56.6 years (standard deviation = 10.7 years); 70.9% of the patients were over 50 years, most were female (72.6%), and the self-declaration of race and/or skin color was mostly “brown” (60.7%). As to schooling levels, 73 patients did not reach high school, and 12% (*n* = 14) were not literate. The mean number of prescribed antihypertensives per patient was 1.87 (standard deviation = 0.87), and 22 respondents (18.8%) reported using three or more medicines to control their blood pressure. The most reported medicines were losartan (*n* = 85) and hydrochlorothiazide (*n* = 49), which 72.6% and 41.9% of patients had used, respectively. The medicines that had these drugs combined were not included in this calculus. Regarding existing comorbidities, 79 patients reported having at least one: diabetes mellitus (*n* = 41; 35%), dyslipidemia (*n* = 39; 33.3%), and cardiovascular complications (*n* = 12; 10.2%).

As to scale adherence classification, 79.5% of patients were considered non-adherent to treatment (*n* = 86). The mean time spent to complete the instrument was 66.6 s. Table 3 shows the study sample characteristics of SMAQ adherence classification.

**TABLE 3:** Study sample characteristics of the antihypertensive pharmacotherapy adherence profile.

Patient characteristic		Total *n* = 117* (%)	SMAQ classification		*p*	MGL classification		*p*
			Adherent n (%)	Non-adherent n (%)		Adherent n (%)	Non-adherent n (%)	
Sex	Male	32 (27.4)	8 (25.0)	24 (75.0)	0.82	13 (40.6)	19 (59.4)	0.82
	Female	85 (72.6)	23 (27.1)	62 (72.9)		29 (34.1)	56 (65.9)	
Schooling	Illiterate	14 (12.0)	4 (28.6)	10 (71.4)	0.90	7 (50.0)	7 (50.0)	0.73
	Kindergarten	4 (4.3)	2 (50.0)	2 (50.0)		1 (25.0)	3 (75.0)	
	Elementary school	17 (14.5)	4 (23.5)	13 (76.5)		4 (36.8)	13 (63.2)	
	Middle school	38 (32.5)	10 (22.6)	28 (77.4)		14 (38.7)	24 (61.3)	
	High school	31 (26.5)	7 (22.6)	24 (77.4)		12 (38.7)	19 (61.3)	
	College	13 (11.1)	4 (30.8)	9 (69.2)		4 (30.8)	9 (69.2)	
Hypertension diagnosis time	6–11 months	3 (2.6)	1 (33.3)	2 (66.7)	0.83	2 (66.7)	1 (33.3)	0.28
	1–4 years	32 (27.6)	10 (31.3)	22 (68.7)		9 (28.1)	23 (71.9)	
	5–9 years	20 (17.2)	4 (20.0)	16 (80.0)		5 (25.0)	15 (75.0)	
	More than 10 years	61 (52.6)	16 (26.2)	45 (73.8)		25 (41.0)	36 (59.0)	
Comorbidity	No	38 (32.5)	7 (18.4)	31 (81.6)	0.17	10 (26.3)	28 (73.7)	0.13
	Yes	79 (67.5)	24 (30.4)	55 (69.6)		32 (40.5)	47 (59.5)	

Pearson’s chi-squared, significance level *p* < 0.05; * in the item “hypertension diagnosis time,” we considered a total of 116 respondents.

### Reliability

The scale reliability was tested by determining its internal consistence with Cronbach’s alpha (Cronbach, 1951).

#### Internal consistence—SMAQ

The Cronbach’s alpha coefficient of the SMAQ scale was 0.631, which is considered satisfactory.

Table 4 shows the values of corrected item–total correlation and Cronbach’s alpha if the item was excluded from this instrument.

**TABLE 4:** Internal consistence of the Brazilian-Portuguese SMAQ scale.

Item	Corrected item–total correlation	Cronbach’s if the item was excluded
1- Do you ever forget to take your medicine?	0.38	0.58
2- Are you careless at times about taking your medicine?	0.26	0.62
3- Sometimes if you feel worse, do you stop taking your medicines?	0.08	0.66
4- Thinking about the last week. How often have you not taken your medicine?	0.42	0.60
5- Did you not take any of your medicine over the past weekend?	0.58	0.52
6- Over the past 3 months, how many days have you not taken any medicine at all?	0.58	0.51

#### Internal consistence—MGL

In addition to the adapted instruments, this study also measured the internal consistence of the MGL scale, Brazilian-Portuguese version, used as criteria validity, and we obtained alpha value 0.627.

#### Criterion validity

The Brazilian-Portuguese version of the MGL pharmacotherapy adherence self-report was used as an external criterion to the concurrent validation process as it is a worldwide instrument and has already been adapted to different health conditions.

#### Concurrent validation—SMAQ

We obtained a positive Spearman’s correlation coefficient of 0.56 between the total score of the adapted SMAQ version and the external criterion of the total MGL, with a significance level of *p* < 0.001. This is considered a satisfactory correlation between the test and the external criterion. The results of total scores and score per item are shown in Table 5.

**TABLE 5:** Total score and score per item correlation of the adapted SMAQ version with the MGL criterion.

		SMAQ variable						
		Total SMAQ	Q1	Q2	Q3	Q4	Q5	Q6
Total MGL	Pearson’s correlation coefficient	0.560	0.487	0.407	0.166	0.248	0.306	0.372
	*p*-value	0.000	0.000	0.000	0.073	0.007	0.001	0.000

### Exploratory factor analysis

For EFA, the initial KMO measure of sampling adequacy was 0.691, and Bartlett’s test of sphericity was significant (χ^2^ = 118.342, *p* < 0.001).

The analysis of factor loadings in the pattern matrix shed light on the relationship between the instrument items and the identified factors related to i) specific medication-taking behaviors and ii) barriers to adherence. Items exhibiting positive loadings in the first factor included items 4, 5, and 6 (factor loadings 0.788, 0.750, and 0.739, respectively). This suggests a positive association between these items and the factor representing specific medication-taking behaviors. Conversely, items 1, 2, and 3 displayed a positive loading in the second factor, indicating a direct association with barriers to adherence (factor loadings 0.579, 0.768, and 0.645, respectively). These findings underscore the multidimensional nature of therapeutic adherence, wherein distinct factors capture different aspects of adherence behavior, including both medication-taking behavior and barriers to adherence.

The EFA revealed eigenvalues exceeding the conventional threshold of 1 for the first two items of the instrument, with values of 2.313 and 1.250, respectively, explaining 59.4% of the variance. These eigenvalues signify substantial variance explained by these items, validating their importance within the measurement construct. While subsequent items displayed eigenvalues below 1, each item elucidated over 7% of the total variance, indicating significant contributions to the construct. Retaining all six items ensures a comprehensive coverage of medication adherence behavior, enhancing the instrument’s validity and sensitivity to diverse patient responses. The decision to retain all items aligns with the instrument’s theoretical framework and intended purpose, facilitating a thorough assessment of medication adherence.

### Known groups’ validation

In this step, we considered that patients with uncontrolled blood pressure are related to low level of antihypertensive pharmacotherapy adherence. The mean values of SBP and DBP were 145.6 mmHg (standard deviation = 19.1) and 88.8 mmHg (standard deviation = 12.0), respectively. Table 6 shows the results of the relationship between SMAQ scale adherence level and blood pressure control. The Pearson’s chi-squared test did not find a statistically significant difference in this association (*p* = 0.56). Thus, it is not possible to conclude that this scale is capable of differentiating patients with good blood pressure control from those with bad control.

**TABLE 6:** Relationship between adherence level and blood pressure (BP) control.

BP control	Classification—SMAQ		Total
	Adherers	Non-adherers	
Controlled BP	13 (11.1%)^d^	31 (26.5%)^b^	44 (37.6%)
Uncontrolled BP	18 (15.4%)^c^	55 (47.0%)^a^	73 (62.4%)
Total	31 (26.5%)	86 (73.5%)	117

a, b, c, d: code to the sensitivity, specificity, and (+) and (−) predictive value calculus.

### Sensitivity, specificity, and predictive values

Concerning blood pressure control, Table 7 shows that the SMAQ scale presented better sensitivity and worse specificity than the MGL standard.

**TABLE 7:** Sensitivity, specificity, positive predictive value, and negative predictive value of each studied instrument.

Instrument	Sensitivity	Specificity	Positive predictive value	Negative predictive value
SMAQ	75.30%	29.50%	63.90%	41.90%
MGL	66.60%	45.20%	68.50%	43.20%

The t-test for equality of means showed a significance level in the relationship between the scores of social desirability of the MC-SDS-BR scale and the adherent behavior for the SMAQ (*p* = 0.019) and MGL (*p* = 0.005) scales. The results are detailed in Table 8.

**TABLE 8:** Social desirability according to adherent behavior classification.

Instrument		Social desirability			*p*
		N	Mean	Standard deviation	
SMAQ	Adherers	31	23.55	3.529	0.019
	Non-adherers	86	21.59	4.796	
MGL	Adherers	42	23.60	3.883	0.005
	Non-adherers	75	21.28	4.726	

## Discussion

Many researchers in the cross-cultural adaptation of instruments have recommended submitting all materials generated throughout an adaptation process to the original developers of the instrument. This practice adds value and ensures the safety and quality of the final product—the adapted instrument (Beaton et al., 2000; LINO et al., 2018). We identified that the back-translation synthesis did not present great discrepancy compared to the original version of the instrument, which shows the quality and consistency of the synthesis version (RA0).

There are some studies of the cross-cultural adaptation and validation of the SMAQ, including those adapted to specific clinical conditions such as patients in hemodialysis, patients with lung cancer and breast cancer, and renal transplanted patients (Ortega Suárez et al., 2011; Oberguggenberger et al., 2012; Theofilou, 2012; Alikari et al., 2017). The Greek version of the SMAQ, modified for patients in hemodialysis, maintained only four items related to pharmacotherapy, changing two points for behavior in the hemodialysis section and including two new Likert-like items related to water restriction and diet compliance, making it a version related to a broad behavior of treatment adherence (Alikari et al., 2017).

In the present study, considering the reliability of the SMAQ scale version, it is notable that the third question shows low corrected item–total correlation (0.08). However, its exclusion does not substantially increase the internal consistency of the instrument (α = 0.661). In addition, this is the only item that is related to treatment safety and potentially adverse effects. A great number of antihypertensive drugs present adverse effects that could interfere in the relationship between patient and prescribed treatment, such as bronchospasm and bradycardia by beta-blockers; dry cough by angiotensin-converting enzyme inhibitors; malleolar edema and headache by calcium channel blockers; sedation, dry mouth, and autoimmune reactions with the agonist of alpha-central action; cramps, fatigue, and sexual dysfunction with diuretics (Malachias, 2016). Excluding the item could affect the recruitment of patients who reflect non-adherent behavior before a possible medicine’s undesirable effect.

Regarding the internal consistence presented by the MGL scale, the result here is similar to that in Morisky et al. (1986) in the original version of this instrument (α = 0.61) when applied to 400 patients with arterial hypertension. The Brazilian version of the MGL, studied by Ben, Neumann, and Mengue (Ben et al., 2012), found alpha equal to 0.73 when tested with 206 patients who also had arterial hypertension diagnosis. Although the literature accepts this alpha value, high internal consistency is generally associated with alpha values above 0.8.

Considering that the studied scales did not achieve a high reliability coefficient, it is important to understand that the Cronbach’s coefficient values are strongly influenced by the instrument’s number of items. The reduced number of items per domain of an instrument may reduce the alpha values, thereby affecting its internal consistency (Souza et al., 2017; Ventura-León and Peña-Calero, 2021).

The original version of the SMAQ scale was based on the MGL scale, maintaining a similar structure in the first three items while introducing three new questions. This alignment likely contributed to the positive outcome observed in the concurrent validity assessment. However, despite this similarity, the third and fourth items showed weak correlation with the external criteria. The low correlation of item 3 may be due to poor understanding of the question. In this item, patients are expected to attribute their perceived worsening in health status to medication, but some patients in our study may have attributed it to other factors not related to medication. In our study, 30% of patients had low literacy levels. Item 4 may be susceptible to memory or reporting biases, with participants possibly failing to accurately recall or underestimating the frequency of missed medication doses, resulting in a weaker correlation with the MGL. Overall, the interpretation of the total score in Pearson’s correlation coefficient suggests a moderately positive association between the MGL and the SMAQ, indicating a tendency for both scores to increase together, though not identically.

The sensitivity of the adapted SMAQ version was similar to that reported by the original authors (72%), who validated it against medication event monitoring systems (MEMS) with people living with HIV. The results of specificity and positive predictive value were considerably superior in the first version (91% and 87%, respectively) (Knobel et al., 2002). However, the Morisky–Green test exhibited similar behavior to that identified by Ben, Neumann, and Mengue (Ben et al., 2012) in patients with uncontrolled blood pressure, demonstrating 61% sensitivity and 36% specificity.

Accordingly, the SMAQ has demonstrated greater potential for correctly detecting patients with uncontrolled blood pressure but is less capable of identifying patients with controlled blood pressure. A similar pattern is observed with the positive and negative predictive values, indicating a higher likelihood of individuals classified as “non-adherers” having uncontrolled blood pressure.

The low specificity results may be attributed to the high social desirability observed within the study sample. “Social desirability” represents the participants’ inclination of biased responding, seeking to give answers that are more socially accepted and denying personal association with socially unacceptable behaviors (Crowne and Marlowe, 1960; Ribas et al., 2004).

The results confirm that patients classified as adherers are prone to present a health behavior with more social desirability, which may suggest a seeming adherence and affect the specificity of self-report parameters of adherence. The desire of social acceptance could skew parameters under evaluation in scientific investigations—a threat to its validity (Poínhos et al., 2008).

Evans (1982) reported the existing positive linear relationship between the MC-SDS score and age, and a negative linear relationship between the MC-SDS and schooling. The general profile of those participating in this study—older individuals, low schooling, a limited socio-economic situation, and predominantly served by the Brazilian Public Health System—tend to influence this result. As public health system users fear some loss in their rights over the integrity of their healthcare, they feel that they need to present socially approved behavior (SILVA, 2015). This observation is corroborated by the study of the Brazilian MC-SDS instrument adaptation, conducted by Ribas, Moura, and Hutz (Ribas et al., 2004), which found a strong correlation of factors as social desirability (*p* < 0.05).

In addition to the higher scores of social desirability, studies suggest that lower schooling and health literacy is associated with inappropriate medicine intake behavior, being a direct or indirect predictor of pharmacotherapy adherence and blood pressure control (Náfrádi et al., 2016; Wannasirikul et al., 2016; Lee et al., 2017; Shi et al., 2019).

In this study, the absence of a direct method of determining adherence during criterion validity testing presents a limitation. Thus, we urge future studies to incorporate MEMS or similar technologies to accurately assess criterion validity. In addition, utilizing MEMS can provide valuable insights into medication-taking behavior and barriers to adherence related to forgetfulness.

Finally, although there are questionnaires already available in Portuguese for evaluating medication adherence, it is imperative in the context of the Brazilian public healthcare system that patient-reported outcome measures be easy to administer and comprehend and are non-proprietary, thus avoiding the need for copyright payments. Additionally, our cross-cultural adaptation process involved the participation of the original SMAQ author, contributing to the semantic equivalence of the translated version. This is expected to benefit healthcare professionals and researchers in their efforts to improve patient care and outcomes in Brazil.

## Conclusion

The cross-cultural adaptation of the SMAQ instrument followed international methodological recommendations, including translation, back-translation, equivalence evaluation, and pretesting. Additionally, both adapted versions were approved by the respective authors of the original instruments, which attests to the credibility of the translation process.

The Brazilian-Portuguese version of the SMAQ scale presented acceptable internal consistence and criterion validity over the MGL scale. This instrument construct validity related to blood pressure control presented a limitation due to its low specificity, which was probably induced by the high social desirability of the sample.

## Data Availability

The raw data supporting the conclusion of this article will be made available by the authors, without undue reservation.

## References

[B1] Alikari V. Matziou V. Tsironi M. Kollia N. Theofilou P. Aroni A. (2017). A modified version of the Greek simplified medication adherence questionnaire for hemodialysis patients. Health Psychol. Res. 5 (1), 6647. 10.4081/hpr.2017.6647 28603780 PMC5452632

[B2] Beaton D. E. Bombardier C. Guillemin F. Ferraz M. B. (2000). Guidelines for the process of cross-cultural adaptation of self-report measures. Spine (Phila Pa 1976) 25 (24), 3186–3191. PMID: 11124735. 10.1097/00007632-200012150-00014 11124735

[B3] Ben A. J. (2011). Confiabilidade e análise de desempenho de dois questionários de avaliação da adesão ao tratamento anti-hipertensivo: teste de Morisky-Green e Brief Medication Questionnaire. 2011. 108 f. Dissertação (Mestrado em Epidemiologia) – Universidade Federal do Rio Grande do Sul. Porto Alegre, Brasil: Universidade Federal do Rio Grande do Sul. Faculdade de Medicina. Programa de Pós-Graduação em Epidemiologia. Available at: http://hdl.handle.net/10183/31878 .

[B4] Ben A. J. Neumann C. R. Mengue S. S. (2012). The Brief Medication Questionnaire and Morisky-Green test to evaluate medication adherence. Rev. Saude Publica 46 (2), 279–289. English, Portuguese. 10.1590/s0034-89102012005000013 22331180

[B5] Beyhaghi H. Reeve B. B. Rodgers J. E. Stearns S. C. (2016). Psychometric properties of the four-item Morisky green levine medication adherence scale among atherosclerosis risk in communities (ARIC) study participants. Value Health 19 (8), 996–1001. Epub 2016 Aug 31. PMID: 27987650; PMCID: PMC5287458. 10.1016/j.jval.2016.07.001 27987650 PMC5287458

[B6] BRASIL (2012). Ministério da Saúde. Conselho Nacional de Saúde. Resolução CNS n. 466, de 12 dezembro de 2012. Aprovar diretrizes e normas regulamentadoras de pesquisas envolvendo seres humanos.

[B7] Brouwers S. Sudano I. Kokubo Y. Sulaica E. M. (2021). Arterial hypertension. Lancet 398 (10296), 249–261. 10.1016/S0140-6736(21)00221-X 34019821

[B8] Cronbach L. J. (1951). Coefficient Alpha and the internal structure of tests. Psychometrika 16 (3), 297–334. 10.1007/BF02310555

[B9] Crowne D. P. Marlowe D. (1960). A new scale of social desirability independent of psychopathology. J. Consult Psychol. 24, 349–354. 10.1037/h0047358 13813058

[B10] Drost E. A. (2011). Validity and reliability in social science research. Educ. Res. Perspect. 38 (1), 105–123.

[B11] Durand H. Hayes P. Harhen B. Conneely A. Finn D. P. Casey M. (2018). Medication adherence for resistant hypertension: assessing theoretical predictors of adherence using direct and indirect adherence measures. Br. J. Health Psychol. 23 (4), 949–966. Epub 2018 Jul 16. PMID: 30014548. 10.1111/bjhp.12332 30014548

[B12] Evans R. G. (1982). Clinical relevance of the Marlowe-Crowne Scale: a review and recommendations. J. Pers. Assess. 46 (4), 415–425. 10.1207/s15327752jpa4604_14 7120022

[B13] Ewen S. Meyer M. R. Cremers B. Laufs U. Helfer A. G. Linz D. (2015). Blood pressure reductions following catheter-based renal denervation are not related to improvements in adherence to antihypertensive drugs measured by urine/plasma toxicological analysis. Clin. Res. Cardiol. 104 (12), 1097–1105. Epub 2015 Aug 26. PMID: 26306594. 10.1007/s00392-015-0905-5 26306594

[B14] Ferreira L. Neves A. N. Campana M. B. Tavares MCGCF (2014). Guia da AAOS/IWH: sugestões para adaptação transcultural de escalas. Aval. Psicol. 13 (3), 457–461.

[B15] Gellad W. F. Thorpe C. T. Steiner J. F. Voils C. I. (2017). The myths of medication adherence. Pharmacoepidemiol Drug Saf. 26 (12), 1437–1441. Epub 2017 Oct 10. PMID: 28994158. 10.1002/pds.4334 28994158

[B16] Guillemin F. Bombardier C. Beaton D. (1993). Cross-cultural adaptation of health-related quality of life measures: literature review and proposed guidelines. J. Clin. Epidemiol. 46 (12), 1417–1432. PMID: 8263569. 10.1016/0895-4356(93)90142-n 8263569

[B17] Judd E. Calhoun D. A. (2014). Apparent and true resistant hypertension: definition, prevalence and outcomes. J. Hum. Hypertens. 28 (8), 463–468. Epub 2014 Jan 16. PMID: 24430707; PMCID: PMC4090282. 10.1038/jhh.2013.140 24430707 PMC4090282

[B18] Kario K. Chia Y. C. Siddique S. Turana Y. Li Y. Chen C. H. (2022). Seven-action approaches for the management of hypertension in Asia - the HOPE Asia network. J. Clin. Hypertens. (Greenwich). 24 (3), 213–223. Epub 2022 Feb 16. PMID: 35172037; PMCID: PMC8925006. 10.1111/jch.14440 35172037 PMC8925006

[B19] Knobel H. Alonso J. Casado J. L. González J. Ruiz I. Kindelan J. (2002). Validation of a simplified medication adherence questionnaire in a large cohort of HIV-infected patients: the GEEMA Study. AIDS 16 (4), 605–613. 10.1097/00002030-200203080-00012 11873004

[B20] Lee C. H. Chang F. C. Hsu S. D. Chi H. Y. Huang L. J. Yeh M. K. (2017). Inappropriate self-medication among adolescents and its association with lower medication literacy and substance use. PLoS One 12 (12), e0189199. 10.1371/journal.pone.0189199 29240799 PMC5730183

[B21] Lino C. R. M. Brüggemann O. M. Souza M. L. Barbosa S. F. F. Santos E. K. A. (2018). The cross-cultural adaptation of research instruments, conducted by nurses in Brazil: an integrative review. Texto & Contexto – Enfermagem 26 (4), 1–11. 10.1590/0104-07072017001730017

[B22] Malachias M. V. (2016). 7th Brazilian guideline of arterial hypertension: presentation. Arq. Bras. Cardiol. 107 (3), 0. PMID: 27819379; PMCID: PMC5319461. 10.5935/abc.20160140 27819379 PMC5319461

[B23] Mokkink L. B. Prinsen C. A. C. Patrick D. L. Alonso J. Bouter L. M. de Vet H. C. W. (2019). COSMIN Study Design checklist for Patient-reported outcome measurement instruments. https://www.cosmin.nl/wp-content/uploads/COSMIN-study-designing-checklist_final.pdf.

[B24] Morisky D. E. Ang A. Krousel-Wood M. Ward H. J. (2008). Predictive validity of a medication adherence measure in an outpatient setting. J. Clin. Hypertens. (Greenwich). 10 (5), 348–354. PMID: 18453793; PMCID: PMC2562622. 10.1111/j.1751-7176.2008.07572.x 18453793 PMC2562622

[B25] Morisky D. E. Green L. W. Levine D. M. (1986). Concurrent and predictive validity of a self-reported measure of medication adherence. Med. Care 24 (1), 67–74. PMID: 3945130. 10.1097/00005650-198601000-00007 3945130

[B26] Náfrádi L. Galimberti E. Nakamoto K. Schulz P. J. (2016). Intentional and unintentional medication non-adherence in hypertension: the role of health literacy, empowerment and medication beliefs. J. Public Health Res. 5 (3), 762. 10.4081/jphr.2016.762 28083523 PMC5206775

[B27] NCD Risk Factor Collaboration (NCD-RisC) (2021). Worldwide trends in hypertension prevalence and progress in treatment and control from 1990 to 2019: a pooled analysis of 1201 population-representative studies with 104 million participants. Lancet 398 (10304), 957–980. 10.1016/S0140-6736(21)01330-1 34450083 PMC8446938

[B28] Nguyen T. M. La Caze A. Cottrell N. (2014). What are validated self-report adherence scales really measuring? a systematic review. Br. J. Clin. Pharmacol. 77 (3), 427–445. PMID: 23803249; PMCID: PMC3952718. 10.1111/bcp.12194 23803249 PMC3952718

[B29] Oberguggenberger A. S. Sztankay M. Beer B. Schubert B. Meraner V. Oberacher H. (2012). Adherence evaluation of endocrine treatment in breast cancer: methodological aspects. BMC Cancer 12, 474. 10.1186/1471-2407-12-474 23066928 PMC3519669

[B30] Oliveira-Filho A. D. Morisky D. E. Neves S. J. Costa F. A. de Lyra D. P. Jr (2014). The 8-item Morisky Medication Adherence Scale: validation of a Brazilian-Portuguese version in hypertensive adults. Res. Soc. Adm. Pharm. 10 (3), 554–561. Epub 2013 Oct 26. PMID: 24268603. 10.1016/j.sapharm.2013.10.006 24268603

[B31] Ortega Suárez F. J. Sánchez Plumed J. Pérez Valentín M. A. Pereira Palomo P. Muñoz Cepeda M. A. Lorenzo Aguiar D. Grupo de Estudio Vatren (2011). Validation on the simplified medication adherence questionnaire (SMAQ) in renal transplant patients on tacrolimus. Nefrologia 31 (6), 690–696. PMID: 22130285. 10.3265/Nefrologia.pre2011.Aug.10973 22130285

[B32] PAHO (2021). Leading causes of mortality and health loss at regional, subregional, and country levels in the Region of the Americas, 2000-2019. ENLACE data portal. Pan American Health Organization. Disponível em: https://www.paho.org/en/enlace/leading-causes-death-and-disability.Acessoem:24/02/2023.

[B33] Poínhos R. Correia F. Faneca M. Ferreira J. Gonçalves C. Pinhão S. (2008). Social desirability and barriers to the accomplishment of the dietary treatment in overweight women. Acta Med. Port. 21 (3), 221–228. Portuguese. Epub 2008 Jul 25. PMID: 18674414. 18674414

[B34] Ribas R. C. Jr Moura M. L. S. Hutz C. S. (2004). Adaptação brasileira da Escala de Desejabilidade Social de Marlowe-Crowne. Aval. Psicol. 3 (2), 83–92.

[B35] Schmidt D. R. C. Dantas R. A. S. (2011). Analysis of validity and reliability of the adapted Portuguese version of Antonovsky's Sense of Coherence Questionnaire among nursing professionals. Rev. Latino-Americana Enferm. 19 (1), 42–49. 10.1590/s0104-11692011000100007 21412628

[B36] Shi S. Shen Z. Duan Y. Ding S. Zhong Z. (2019). Association between medication literacy and medication adherence among patients with hypertension. Front. Pharmacol. 10, 822. 10.3389/fphar.2019.00822 31396088 PMC6664237

[B37] Silva J. C. S. (2015). Atendimento na rede de atenção à saúde: a percepção de usuárias em Goiânia-GO. 2015. 199 f. Dissertação (Mestrado Profissional em Ensino na Saúde) - Universidade Federal de Goiás. Goiânia.

[B38] Souza A. C. Alexandre N. M. C. Guirardello E. B. (2017). Psychometric properties in instruments evaluation of reliability and validity. Epidemiol. Serv. Saude 26 (3), 649–659. 10.5123/S1679-49742017000300022 28977189

[B39] Taherdoost H. Sahibuddin S. Neda J. (2014). “Exploratory factor analysis; concepts and theory,” in Advances in applied and pure mathematics. Mathematics and computers in science and engineering series. Editor Balicki J. (Kuala Lumpur, Malaysia: Universiti Teknologi Malaysia), 27, 375–382. 978-960-474-380-3. hal- 02557344. Available at: https://ssrn.com/abstract=4178683 .

[B40] Theofilou P. (2012). Results from the translation and cultural adaptation of the Greek Simplified Medication Adherence Questionnaire (GR-SMAQ) in patients with lung cancer. J. Clin. Trials 3 (2). 10.4172/2167-0870.1000133

[B41] Unni E. J. Farris K. B. (2015). Development of a new scale to measure self-reported medication nonadherence. Res. Soc. Adm. Pharm. 11 (3), e133–e143. Epub 2009 Oct 9. PMID: 21272524. 10.1016/j.sapharm.2009.06.005 21272524

[B42] Ventura-León J. Peña-Calero B. N. (2021). The world should not revolve around Cronbach's alpha ≥ 70. Adicciones 33 (4), 369–372. English, Spanish. 10.20882/adicciones.1576 33338249

[B43] Wannasirikul P. Termsirikulchai L. Sujirarat D. Benjakul S. Tanasugarn C. (2016). Health literacy, medication adherence, and blood pressure level among hypertensive older adults treated at primary health care centers. Southeast Asian J. Trop. Med. Public Health 47 (1), 109–120. PMID: 27086432. 27086432

[B44] Wild D. Grove A. Martin M. Eremenco S. McElroy S. Verjee-Lorenz A. (2005). Principles of good practice for the translation and cultural adaptation process for patient-reported outcomes (PRO) measures: report of the ISPOR task force for translation and cultural adaptation. Value Health 8 (2), 94–104. 10.1111/j.1524-4733.2005.04054.x 15804318

[B45] Williams B. Mancia G. Spiering W. Agabiti Rosei E. Azizi M. Burnier M. (2018). 2018 practice guidelines for the management of arterial hypertension of the European society of hypertension and the European society of cardiology: ESH/ESC task force for the management of arterial hypertension. J. Hypertens. 36 (12), 2284–2309. Erratum in: J Hypertens. 2019 Feb;37(2):456. PMID: 30379783. 10.1097/HJH.0000000000001961 30379783

[B46] Zakariya Y. F. (2022). Cronbach's alpha in mathematics education research: its appropriateness, overuse, and alternatives in estimating scale reliability. Front. Psychol. 13, 1074430. 10.3389/fpsyg.2022.1074430 36619096 PMC9813591

[B47] Zayed H. S. Medhat B. M. Seif E. M. (2019). Evaluation of treatment adherence in patients with Behçet's disease: its relation to disease manifestations, patients' beliefs about medications, and quality of life. Clin. Rheumatol. 38 (3), 761–768. Epub 2018 Oct 26. PMID: 30367312. 10.1007/s10067-018-4344-3 30367312

